# Achieving COVID‐19 herd immunity in Rwanda, Africa

**DOI:** 10.1002/puh2.75

**Published:** 2023-03-17

**Authors:** Theogene Uwizeyimana, Emery Manirambona, Shuaibu Saidu Musa, Emmanuel Uwiringiyimana, Darius Bazimya, Kedest Mathewos

**Affiliations:** ^1^ School of Medicine, University of Global Health Equity Kigali Rwanda; ^2^ Global Health Focus Africa Kigali Rwanda; ^3^ Global Health Focus Africa Gitega Burundi; ^4^ Department of Nursing Science Ahmadu Bello University Zaria Nigeria; ^5^ University of Rwanda – College of Medicine and Health Sciences Kigali Rwanda

**Keywords:** Africa, COVID‐19, herd immunity threshold, pandemic, Rwanda, vaccination, vaccines

## Abstract

The COVID‐19 pandemic has caused significant disruption to global healthcare systems, including Rwanda's. Rwanda has taken measured actions in response to the pandemic using a multisectoral and one‐government approach. The introduction of the COVID‐19 vaccine boosted the hope of many people but has left debates on the measure of the population required to attain herd immunity. Herd immunity threshold (HIT) was introduced to track progress toward containing COVID‐19. HIT represents the number of people with long‐term immunity from COVID‐19. World Health Organization (WHO) established population vaccination targets for all countries worldwide, which included 40% coverage of the entire population by the end of 2021 and 70% by June 2022. In this paper, we discussed the efforts and progress that have been made so far toward achieving herd immunity in Rwanda.

## INTRODUCTION

It has been more than 2 years since the COVID‐19 pandemic wreaked havoc on healthcare systems globally, including that of Rwanda. Since the report of the first case of COVID‐19 on 14 March 2020 in the country, Rwanda set up a national committee comprising representatives from various ministries to combat the disease outbreak in the country using a multisectoral and one‐government approach [1]. To contain COVID‐19, Rwanda has deployed various strategies, including massive and intensive health information on COVID‐19 and the provision of COVID‐19 vaccines [1]. The introduction of the COVID‐19 vaccine boosted the hope of many people but left questions on how many people need to be vaccinated for everyone to go back to their pre‐pandemic lives or to lift stringent COVID‐19 preventive measures. Thus, herd immunity threshold (HIT), which is the number of population that get long‐term immunity from COVID‐19 (through vaccination or previous infection), was introduced. HIT is required to stop new COVID‐19 infections and transmission. It was introduced to help different stakeholders monitor their progress toward containing the virus. However, studies have identified that all COVID‐19 variants do not have the same HIT, with the original SARS‐CoV‐2 variant having a range between 60% and 75%, omicron and delta variants with a higher threshold of 80% and above [2]. Despite these, the World Health Organization (WHO) has established population vaccination targets for all countries worldwide. This included 40% coverage of the entire population by the end of 2021 and 70% by June 2022 [3]. Rwanda has been so keen to achieve these global targets, and the question remains whether HIT has been achieved or not. In this paper, we aim to discuss the efforts and progress that have been made so far toward achieving herd immunity in Rwanda.

## PREVENTIVE MEASURES, VACCINE ROLLOUT, AND HERD IMMUNITY

Since the occurrence of the first COVID‐19 case, Rwanda has established regular cabinet meetings to review COVID‐19 precautionary measures and has implemented a series of COVID‐19 preventive measures. These measures include, but are not limited to, school and church closures, curfews, regular handwashing, mandatory mask wearing while in public, lockdowns, and prohibition of mass gatherings and unnecessary travels [1]. In addition, numerous interventions were adopted to fight the pandemic, and that included community awareness and education through different channels such as through state television and radio, social media, as well as using drones with speakers [1, 4]. Anti‐epidemic robots and mobile laboratories were used to support health workers in monitoring COVID‐19 patients and increase testing capacity, hence strengthening the rapid response to the pandemic [1]. Based on an analysis of the country's pandemic situation at the national and local levels, the COVID‐19 precautionary measures are constantly revised, adapted, and implemented.

Rwanda's population is 12,952,209, and the country has so far conducted over 5,910,710 COVID‐19 tests and registered over 132,560 COVID‐19 cases and 1467 deaths as of 23 February 2023 [5]. The country has experienced a decrease, on the average of new cases per 7 days from 477 cases in January 2022 to 11 cases in February 2023 [6]. Following this significant decrease in new cases, some preventive measures were relaxed. Ban on public gatherings and events were lifted, businesses and land borders were reopened, restrictions on public transport were removed, and curfew hours were lifted, among others. Although preventive measures have been lifted, COVID‐19 vaccination programs have been enforced, and COVID‐19 vaccination certificates are being asked to allow access or to be able to attend many public gatherings.

The country has approved six COVID‐19 vaccines for use in its COVID‐19 vaccination program. These are Pfizer/BioNTech, Moderna, Oxford/AstraZeneca, Janssen (Johnson & Johnson), Gamaleya, and Sinopharm (Beijing). It has also allowed three clinical trials of COVID‐19 vaccines, including Novavax, Inovio, and Janssen (Johnson & Johnson) [7]. The COVAX initiative, a platform for accelerating the development and equitable distribution of COVID‐19 vaccines to all countries worldwide [8], has supported the country's vaccine program. This was further strengthened by the African Vaccine Acquisition Trust (AVAT), which was primarily created to support African countries specifically in securing enough vaccines for their population. Furthermore, bilateral collaborations and direct purchases from vaccine manufacturers have increased the country's COVID‐19 vaccine supply [4]. The vaccines have been distributed at COVID‐19 vaccination units at many health centers and public hospitals. Mobile clinics have been deployed in public places to facilitate access to the COVID‐19 vaccination program and to boost the vaccination coverage. These efforts compliment the COVAX recommendation of having ambitious targets for vaccination program implementation, as well as expanding the national strategies of enforcing vaccination coverage by all countries worldwide to address global vaccine inequity.

Rwanda received the first shipment of 1000 doses of COVID‐19 vaccine from Moderna in February 2021, followed by another shipment of 240,000 doses of AstraZeneca‐Oxford and 102,960 doses of Pfizer/BioNTech vaccines from the COVAX initiative in March 2021 [9]. Prior to the first vaccine shipment, numerous preparations were made, including the acquisition of ultracold freezers and containers for vaccine storage and distribution, as well as the training of various personnel and preparation of facilities to allow for smooth vaccine rollout [9]. The Rwandan Ministry of Health distributed all of the vaccines to district hospitals and health centers, kicking off the first phase of mass vaccination. It was aimed at the high‐risk population that included refugees and the elderly in prison populations. This exercise was carried out in conjunction with other COVID‐19 prevention measures that were already in place.

Globally, over 69.6% have been vaccinated with the first dose of the COVID‐19 vaccine as of 23 February 2023, but only 27.6% of them are from low‐income countries [10]. Rwanda, a low‐income country, however, has made a remarkable progress in vaccinating over 81.6% of its population following the initial COVID‐19 vaccination protocol [6]. The initial vaccination protocol comprised two doses of COVID‐19 vaccines plus a booster dose (third dose). Rwanda achieved this by striving to reach WHO vaccination targets that called all countries worldwide to vaccinate at least 40% of their population by the end of 2021 and 70% by mid‐2022 [3]. According to WHO, the vaccination coverage of 70% is believed to be HIT for COVID‐19. In June 2022, around 9092,255 (70.19%) of the Rwandan population were vaccinated with at least 1 dose of the COVID‐19 vaccine, 8601,838 (66.41%) with a second dose, whereas 4840,072 (37.36%) with a booster dose (third dose) [5].

According to the 2019 Wellcome Trust report, 97% of Rwandans have confidence in their country's healthcare system [11]. This trust is believed to have played a crucial role in increasing Rwanda's vaccination rate. Prior to the COVID‐19 pandemic, Rwanda had a well‐functioning immunization program and has made tremendous progress that includes routine immunization services where 96% of under‐five children receive all basic vaccinations, whereas 84% receive age‐appropriate vaccinations [12]. This scenario helped in the employment of other strategies/efforts in addition to already established COVID‐19 precautionary measures to improve the response and vaccination coverage (see Table 1).

**TABLE 1:** Rwandan strategies/efforts to improve COVID‐19 vaccination coverage.

Strategies/efforts	Description	Implication
Vaccination at market places/public gatherings	Rwanda established vaccination stations in various locations, including markets, bus terminals, and other significant public gathering places. In each location, health volunteers responsible for educating the public about the importance of immunizations were stationed at the entrance and screened the vaccination status of all individuals entering the site before directing them to a tent where a trained nurse was administering vaccines	This strategy has increased vaccination awareness, decreased vaccine hesitancy among the community due to their busy lifestyles, and enabled mass vaccination
Vaccination at health centers	Rwanda established a decentralized vaccination program and provided health centers (which are located in close proximity to communities) with the resources required to implement the program. The ultracold refrigerators were distributed to health centers, and staff members were trained to assist them in having more accomplishments	Providing vaccines at the health center level instead of just district hospitals increases geographic access to the vaccines. This allowed vulnerable communities in very remote areas to receive vaccines thus improving equitable access
Mobile clinics	Rwanda has deployed the use of mobile clinics to improve vaccination coverage. A van that circulates on different streets of Kigali (the capital city of Rwanda) is used mainly to increase the uptake of booster doses. This van comes in addition to a door‐to‐door mass campaign to increase vaccination awareness and to remind the community to take all required doses	This strategy is aimed at bringing vaccines very close to the people, particularly those with busy schedules. This allowed adults who had yet to take booster doses after 3 months of their second dose to get one while continuing their businesses

These efforts have resulted in a remarkable progress in the fight and containment of the spread of the virus, thus reducing the mortality rate and making Rwanda one of the first among low‐income countries to achieve the WHO COVID‐19 vaccine target. The Rwandan government invested much in the implementation of these efforts. Thus, it was not surprising that in the week of 02 October 2022, the positivity rate (the percentage of all positive reported tests) was 0.07% with the weekly incidence rate being one in 100,000 people. This was consistent with the previous week's positivity rate of 0.10%, with a similar weekly incidence rate [5]. This significant decrease in transmission shows that the needed threshold for herd immunity has been achieved, but more studies need to be done to test the hypothesis. Despite the introduction of the second COVID‐19 booster dose (fourth dose) on 08 August 2022 in Rwanda [5], the current vaccination coverage achievements and the pandemic status are promising and can serve as a lesson for other low‐income countries that still lag behind to meet their vaccination targets.

## CONCLUSION

Rwanda has made the remarkable progress in vaccinating the majority of the population against the COVID‐19 pandemic. However, despite this progress, it is important to keep the speed in order to fully control and prevent the COVID‐19 pandemic. Rwanda should not stop the fight against the disease given that the COVID‐19 may mutate to other new variants. The country's COVID‐19 surveillance activities should remain vital to ensure that the country is safe and protected. Increasing vaccination coverage would play an essential role in achieving further herd immunity. Strong political commitment and proper allocation of resources should be the core for disease prevention in all countries, especially those from low‐ and middle‐income settings.

## CONFLICT OF INTEREST STATEMENT

The authors declare no conflicts of interest.
